# Supplementary material for: Association between oral microbiome and seven types of cancers in East Asian population: a two-sample Mendelian randomization analysis
Source: Front Mol Biosci. 2023 Nov 21;10:1327893. doi: 10.3389/fmolb.2023.1327893 (PMC10702768; doi:10.3389/fmolb.2023.1327893)
Supplement: Supplementary file 1 [file DataSheet1.ZIP › Supplementary Materials/MR plots for tongue/tongue═╝/Ovarian cancer/pheno.2280_to_ovarian cancer_funnel.pdf]

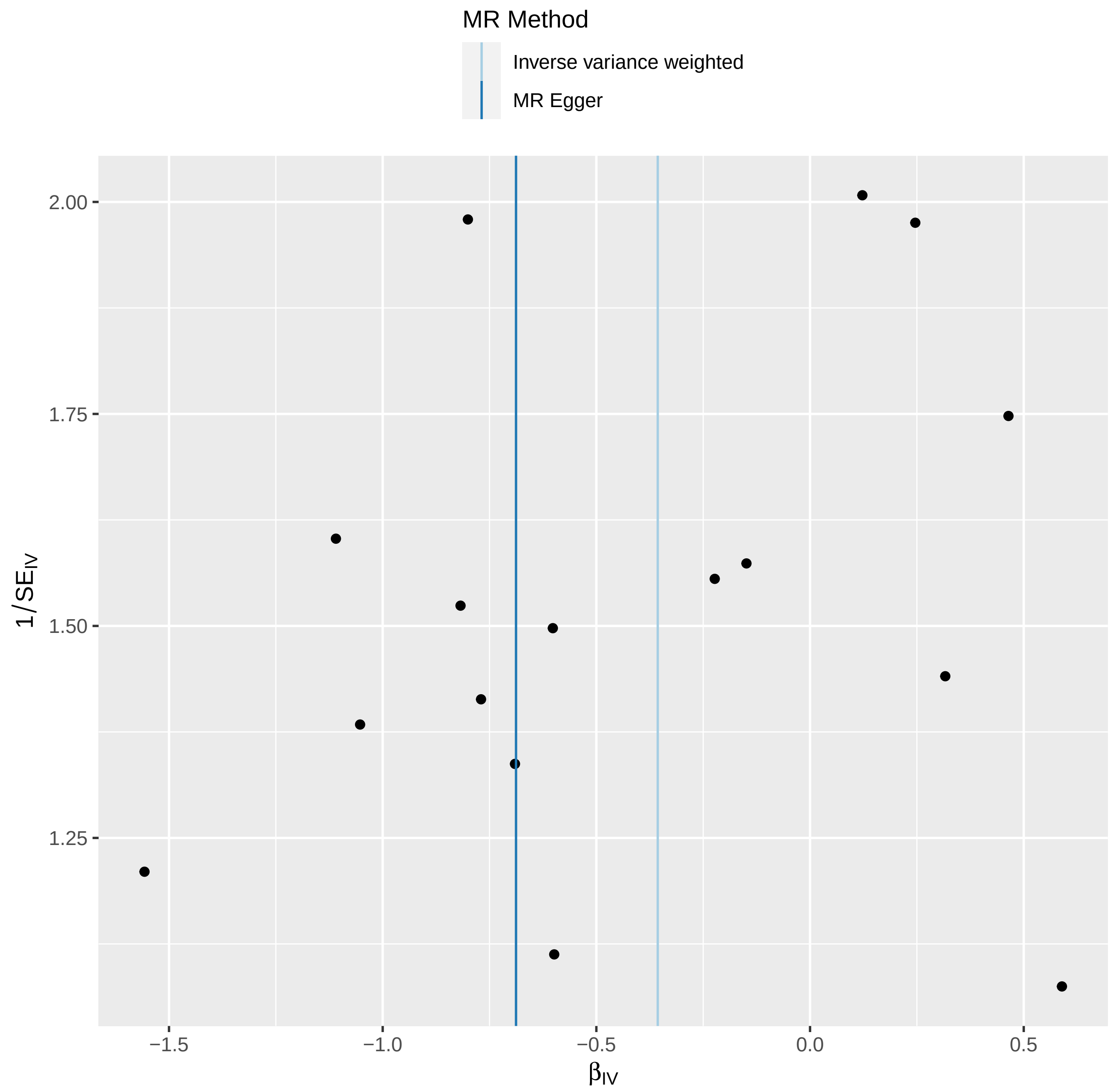
MR Method
Inverse variance weighted
MR Egger
2.00
1.75
1.50
1.25
1/SE_IV
−1.5
−1.0
−0.5
0.0
0.5
β_IV
